# Vital Force

**Published:** 1873-01

**Authors:** Geo. Watt


### Vital Force.

BY GEO. WATT.

*Read Before the Ohio State Dental Society.*

A lengthy disquisition on the correlation of forces might, with profit, precede the discussion of this, but neither time nor the individualized portion of this force behind the pencil will admit. Suffice it is to say that vital force, in our present state of existence, is the result of chemical force, or at least of chemical action. It is not proposed here to discuss the power of pure or disembodied spirits, but to consider man as we find him, composed of a physical body and a spiritual mind, each influencing the other as to development, manifestation, progress, etc.; the mind in this state of existence being able to act or give manifestation only by and through the body.

From the dawn of existence a battle rages in the human body, and ceases only when what is called death closes the conflict. The combatants are nutrition and respiration. The one builds up the body; the other burns it down; and in this conflict all that we know of vital force is developed.

Nutrition, or the builder, *creates* nothing. It appropriates and assimilates to its uses matter already in existence. And as matter is always governed by laws, it must use it in obedience to law. It is as much bound by chemical as by physical laws; and who would think, for a moment, of nutrition being carried on regardless of gravitation, solubility, hardness, softness and the like? It is an error far to common to regard vital force as something wholly above and independent of chemical action; and those who do not go quite so far, speak of vital force *modifying* chemical action as if such a thing were unique in nature, forgetting that gravitation, cohesion, etc., also modify it. All this amounts to is that matter is obedient to law—that each law modifies all the rest, or in other words, that they all harmonize, which is but a truism, for they are all laws, not of nature, but of nature's God.

Nutrition then builds up our bodies in obedience to chemical affinity, and all the other laws which govern the materials composing it, mind, or will-power, being one of them.

Leaving to the general physiologist the details of the process of nutrition, let us pass from the builder to the destroyer.

Respiration is the function by which oxygen is conveyed directly to the blood and oxydized matter directly from it. It has also other incidental uses not to be noticed here. In nutrition oxygen is extensively used, as it is a prominent constituent of organic bodies; but the oxygen introduced by respiration is not assimulated—does not help to build up tissue, but always to destroy it.

The red corpuscles receive and carry the oxygen taken in by respiration. Their ability to do so seems to depend on two of their component substances, iron and "phosphorized fatty matter." The special office of iron in this function was discussed in a paper read before this society two years ago, in which it is suggested that this element cannot carry more oxygen than is used in the functions of organic life, leaving voluntary motion and thought to be still accounted for. The phosphorized matter being so highly combustible (oxidizable) it has been thought marvelous that the corpuscle can carry free oxygen without being itself destroyed by oxidation. But a little observation will find analogous cases. Iron has an affinity for active oxygen strong enough to decompose water, and even to take fire in the atmosphere if divided to the proper degree of minuteness. But iron may be suspended in an atmosphere of pure oxygen for months without even rusting. The explanation is that oxgen has an active and a passive condition, in the latter of which it manifests but little, if any affinity for anything. Iron also has its two conditions. When passive it will remain bright, even in a solution of nitric acid. The same is true of phosphorus, which in its passive state is not combustible, in the ordinary acceptation of the term.

Now, let oxygen have its passive state, and we are no longer surprised that the corpuscle can carry it, esp ecially if its phosphorus be also passive.

Oxygen is changed from active to passive in several ways. As a constituent of the atmospere the great bulk of it is passive. If otherwise, its local effect on the air passages and the skin would be disagreeable and disastrous, as is seen in the prevalence of catarrh and influenza when ozone, which is only active oxygen, is especially noticeable in the atmosphere.

Electricity passing through passive oxygen renders a portion of its active. This accounts for ozone, or active oxygen, in the atmosphere.

When vapor is condensed the heat of vaporization or part of it at least, is changed to electricity. It is in this way that the formation of clouds causes electric phenomenon. When the atmosphere for a time has been unfavorable to the conduction of electricity the clouds are seen with well-defined borders, which indicates a great disturbance of the electric equilibrium. In this condition myriads of infinitesimal sparks of electricity pass through the air, rendering a part of its oxygen active, or as commonly expressed, forming ozone. A part of the ozone is neutralized by local action along the air cells of the lungs, and there obeying the law of gaseous diffusion passes to the blood, and if not exhausted by changing the protoxyed of iron to the sesquioxyed the residue probably remains active, and therefore not obedient to the will, thus explaining the involuntary twitchings, the fever, irritability, and general unrest incident to living in such an atmosphere.

In the paper already referred to I called the oxygen not associated with the iron in the corpuscle, "oxygen of reserve."* For the best health this should be passive till rendered active by the will, as the corpuscle gives it up in obedience to the same will power.

By the oxidation of tissue, as here feebly described, we have the manifestation of vital force both voluntary and involuntary. That the voluntary and involuntary may have proper relations to each other, and to the ends for which we are created, several things are necessary. The oxidized matter must be duly carried out of the system. This is done in part by the skin, kidneys, etc., but mainly by expiration. In other words the blood must not be forced to carry effete matter. The corpuscle must have its due amount of iron and phosphorized matter, and to retain these to proper vital connection of course all its other constituerts must be normal.

It is almost impossible to conceive the voluntary power of a well-formed human body where the corpuscles carry their full proportion of oxygen of reserve in complete subordination to the will. The oxygen of reserve is laid in while we sleep, and many things interfere with its proper accumulation. And even if the corpuscles have a fair supply of it, they may not be able to hold it in obedience to the will, as is manifested by restleness of body and fretfulness of mind, the oxidation of tissues being fitful and irregular, and the manifestation of life force being equally so. Now, if by any means the corpuscles can be rendered able to hold the reserve oxygen in subordination to the will this irregularity of life force will cease. Tea and coffee are used for this purpose, it being common for authors to state that they "arrest the waste of tissue," without giving us a hint as to how they do it. Opium, and perhaps tobacco, have a similiar effect, while alcohol seems to act in the opposite direction, setting free much of the oxygen which should remain in reserve, giving rise to irregularity of action both of mind and body, resulting in the exhaustion observable at the close of a drunken debauch.

The presence of alcohol in the blood may also cause irregular manifestations of life force in another way. Even if the reserve oxygen in the corpuscles obey the will it is likely to act on the alcohol as on anything else; for do we not know that alcohol is highly combustible—that it has a strong affinity for active oxygen? Let us suppose that a corpuscle, in obedience to the will, liberates oxygen to combine with some tissue in the brain for the purpose of developing thoughts of paternal kindness, but the oxygen finds this tissue soaked in alcohol, and combines with both; is it strange if the father *thinks* of his child in obedience to the oxidation of the tissue, and *kills* it in obedience to the oxidation of the alcohol? All that the will has to do with such a transaction is in the previous taking of the alcohol, and in thinking who to kill. And this explains why the *will* of the habitual "moderate drinker" can give him no aid in arresting his progress toward the career of the confirmed drunkard. The only way he can produce action of any kind by will power is by rendering active the reserve oxygen of the corpuscles and setting it free, when, if it finds alcohol at its point of freedom, instead of the normal tissue the action is abnormal—not in obedience to the will, not in harmony with, but wholly outside of physiology.

The reader may regard this article as somewhat dogmatical. I have only given my *thoughts* and *opinions* without the sycophantic "I opine," or "it seems" in each paragraph.

The reader can think for himself, if these thoughts do not suit him.

